# Synthesis of g-C_3_N_4_-Decorated ZnO Porous Hollow Microspheres for Room-Temperature Detection of CH_4_ under UV-Light Illumination

**DOI:** 10.3390/nano9111507

**Published:** 2019-10-23

**Authors:** Min Xiao, Yanwei Li, Bo Zhang, Guang Sun, Zhanying Zhang

**Affiliations:** School of Materials Science and Engineering, Cultivating Base for Key laboratory of Environment-Friendly Inorganic Materials in University of Henan Province, Henan Polytechnic University, Jiaozuo 454000, China; xm2019626@163.com (M.X.); zhb@hpu.edu.cn (B.Z.); zhangzy@hpu.edu.cn (Z.Z.)

**Keywords:** hollow microspheres, g-C_3_N_4_/ZnO, CH_4_, UV-light illumination, gas sensor

## Abstract

UV light-assisted gas sensors based on metal oxide semiconductor (MOS) have attracted much attention in detecting flammable and explosive gases at room temperature. In this paper, graphite-based carbon nitride (g-C_3_N_4_) nanosheets-decorated ZnO porous hollow microspheres (PHMSs) with the size about 3~5 μm in diameter were successfully synthesized by annealing the solvothermally-synthesized Zn_5_(CO_3_)_2_(OH)_6_ PHMSs together with g-C_3_N_4_. The synthesized samples were characterized by XRD, SEM, TEM, FT-IR and XPS. The results indicated that the prepared g-C_3_N_4_/ZnO PHMSs were constructed by numerous loosely stacked ZnO nanoparticles of 20~30 nm in diameter. Gas sensing tests indicated that under UV light (365~385 nm) illumination, the sensors fabricated with g-C_3_N_4_/ZnO HPMSs showed an enhanced response and faster response speed than the pure ZnO counterpart at room temperature. In addition, the g-C_3_N_4_/ZnO sensor also exhibited good repeatability and long-term stability for CH_4_ detection.

## 1. Introduction

As active materials of solid-state gas sensors, metal oxide semiconductor (MOS) has been recognized a promising candidate for develop light-assisted low temperature sensor for the detection of flammable and explosive gases [1,2,3,4,5]. Methane (CH_4_), as a colorless and odorless fuel gas, has been widely used in various industries, as well as human daily life. However, owing to its flammable nature, the leakage of CH_4_ can endanger our environment by causing fire and explosion accidents. Therefore, developing safe and rapid methods for detecting CH_4_ is greatly desired. ZnO, as a cheap and easily available MOS material with a band gap of 3.4 eV, is a promising material for developing light-activated gas sensors that can work at a low temperature condition. Espid et al. studied the photo-responsive performance of ZnO/In_2_O_3_ sensors for detecting NO_2_ under UV LED irradiation at room temperature [6]. They found that the sensing process of composite materials relies on the irradiation of ultraviolet light, which can affect the response time of the sensors by changing the UV flux. Gong et al. fabricated the ZnO nanowires/optical fiber hybrid structure with responding to low concentration ethanol at ppb-level and achieved lower temperature sensing under UV light activation, the combination of UV radiation and ZnO is an effective way to develop high performance gas sensors [7]. Da Silva et al. synthesized ZnO-SnO_2_ heterojunctions for the room temperature photoluminescence-based ozone gas sensing, these ZnO-SnO_2_ heterojunctions were able to detect ozone with the concentration as low as 20 ppb [8]. The results of above reports indicate that UV irradiation is a promising way to achieve low temperature detection and improve gas sensing performance of ZnO sensors. However, there are few reports on the design and synthesis of ZnO based light-activated gas sensing materials for CH_4_ detection. 

Two-dimensional (2D) graphite-based carbon nitride (g-C_3_N_4_) is a metal-free polymer n-type semiconductor. The unique electrical, optical, and physiochemical properties of g-C_3_N_4_ makes the g-C_3_N_4_-based material a new multifunctional platform for developing high performance MOS sensors [9,10]. Presently, g-C_3_N_4_-based gas sensors have attracted an increasing interest. Zhang et al., synthesized α-Fe_2_O_3_/g-C_3_N_4_ nanocomposites by a hydrothermal and pyrolysis method, and the α-Fe_2_O_3_/g-C_3_N_4_ sensor demonstrates a better gas sensing properties than the pure α-Fe_2_O_3_ and g-C_3_N_4_ [11]. Zhai et al. studied the UV light-assisted g-C_3_N_4_/ZnO composites for ethanol sensing characteristics at room temperature, which indicates that the content of g-C_3_N_4_ in the g-C_3_N_4_/ZnO composites has a great influence on the gas sensing performance [12]. These reports indicate that g-C_3_N_4_ can be an efficient material for improving the performance of ZnO MOS gas sensors. However, based on the excellent photocatalytic ability of g-C_3_N_4_, the g-C_3_N_4_-based materials are mostly used in energy and environmental photocatalysis applications, such as water splitting, environmental remediation and carbon dioxide reduction [13,14,15]. The design of high performance light-activated MOS gas sensors by utilizing g-C_3_N_4_ as sensitizer is still in the nascent stage. 

In this paper, ZnO PHMSs decorated with g-C_3_N_4_ nanosheets were successfully prepared by annealing solvothermally-synthesized Zn_5_(CO_3_)_2_(OH)_6_ HMSs together with g-C_3_N_4_. The prepared samples were characterized with XRD, SEM, TEM, FT-IR and XPS. The results proved the successful decoration of g-C_3_N_4_ nanosheets on the porous shell of the ZnO PHMSs. The gas sensing properties of the prepared g-C_3_N_4_/ZnO composites were investigated. It was found that after decorating ZnO PHMSs with g-C_3_N_4_ nanosheets, the sensors showed a faster response speed and an enhanced response under UV-light illumination. 

## 2. Materials and Methods

### 2.1. Materials 

Urea (CH_4_N_2_O, 99%) and zinc acetate [Zn(CH_3_COO)_2_·2H_2_O, 99.0%] were purchased from Kermel (Kermel, Tianjing, China), citric acid (C_6_H_8_O_7_·H_2_O, 99.5%) and absolute ethyl alcohol (C_2_H_6_O, 99.7%) were purchased from Hongyan (Hongygan, Tianjing, China), distilled water was used throughout the experiments, all chemical reagents are analytical and are used without further purification. 

### 2.2. Synthesis of g-C_3_N_4_/ZnO PHMSs 

The synthesis of g-C_3_N_4_ is similar to our previous method [16]. 100 g urea was uniformly placed in a crucible and heated in a muffle furnace at 250 °C for 1 h in an air atmosphere at atmospheric pressure, 350 °C for 2 h, and finally 550 °C for 2 h, with a heating rate of 2 °C min^−1^, the yellow powder of g-C_3_N_4_ was received after cooling to room temperature. 

**Figure 1:**
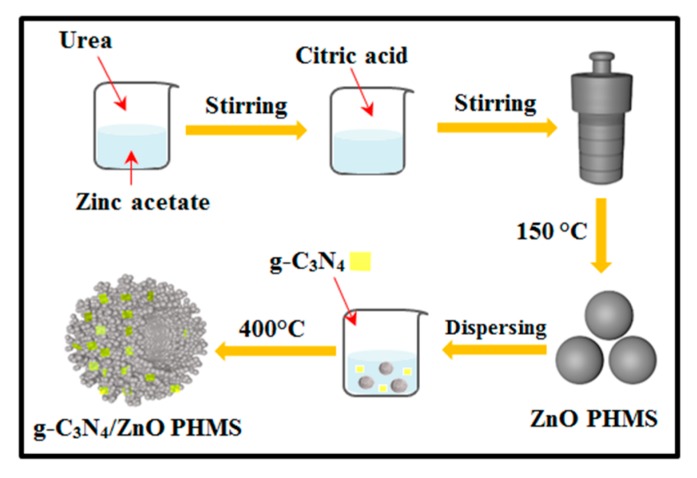
Schematic illustration of the synthesis process for g-C_3_N_4_/ZnO PHMSs.

Zn_5_(CO_3_)_2_(OH)_6_ was prepared by a solvothermal method. In a typical procedure, as illustrated in Figure 1, a mixed solution was obtained by adding 10 mL ethanol solution of urea (1.2 mol/L) and 5 mL ethanol solution of citric acid (0.12 mol/L) into 30 mL aqueous solution of Zn(CH_4_COO)_2_ (0.1 mol/L), in sequence. After stirring for 0.5 h, the mixed solution was transferred into a 50 mL Teflon-lined autoclave and maintained at 150 °C for 10 h. The precipitate was collected by centrifugation, washed with distilled water and ethanol, and dried in air at 60 °C, to obtain the Zn_5_(CO_3_)_2_(OH)_6_ precursor. 

In order to synthesize g-C_3_N_4_/ZnO PHMSs, a desired amount of g-C_3_N_4_ was ground in an agate mortar and then dispersed in 20 mL of ethanol solution. After adding 250 mg of as-prepared Zn_5_(CO_3_)_2_(OH)_6_, the mixture solution was stirred at room temperature for 0.5 h and then dried at 70 °C to remove the solvent. The obtained powder mixture was then annealed at 400 °C in N_2_ atmosphere for 1.5 h to obtain the final g-C_3_N_4_/ZnO samples. By adjusting the using amount of g-C_3_N_4_, g-C_3_N_4_/ZnO samples with the g-C_3_N_4_ content of 3 wt.%, 5 wt.%, 8 wt.%, and 10 wt.% were prepared, which were denoted as CNZO-3, CNZO-5, CNZO-8, and CNZO-10, respectively. Pure ZnO was also prepared by directly annealing the Zn_5_(CO_3_)_2_(OH)_6_ precursor at the same condition.

### 2.3. Characterizations

The morphology and microstructure of prepared samples were observed by field-emission scanning electron microscopy (SEM, JEOL, JSM-6390LV, Tokyo, Japan) and transmission electron microscopy (TEM, JEOL, JEM-2100, Tokyo, Japan). The crystal phase of samples was analyzed by X-ray diffraction (XRD, Bruker/D8-Advance diffractometer, Brukerplc, Billerica, MA, USA) with Cu-Kα radiation in a scanning range of 10–80° (2θ). Surface chemical element analysis was characterized by using X-ray photoelectron spectroscopy (XPS, Thermo ESCALAB 250XI electron spectrometer, Waltham Mass, Waltham, MA, USA) with Al-Kα radiation, the C 1s peak was fixed at a binding energy of 284.6 eV. Fourier Transform Infrared Spectrometer (FT-IR, TENSOR27, Brukerplc, Billerica, MA, USA) with a resolution of 1 cm^−1^. The Brunauer Emmett Teller (BET) specific surface areas of the prepared samples were measured by nitrogen adsorption on a Quantachrome Autosorb-iQ sorption analyzer (Quantachrome, Boynton Beach, FL, USA).

### 2.4. Gas Sensor Fabrication and Analysis

**Figure 2:**
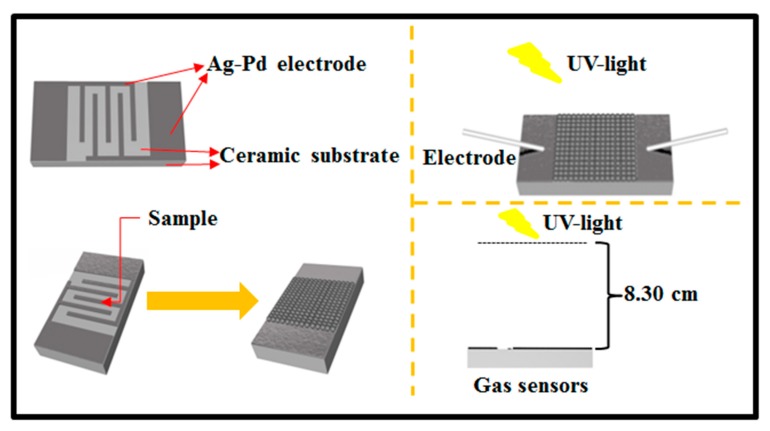
Schematic illustration of the synthetic steps for gas sensor.

The fabrication process of sensors is shown in Figure 2. In detail, a proper amount of as-prepared sample was mixed with in a few drops of deionized water to obtain slurry, which was carefully coated onto an Al_2_O_3_ ceramic substrate (13.4 × 7 mm) with interdigitated Ag-Pd electrodes and heat-treated at 60 °C for 24 h to obtain a resistance-type sensor. 

The light-activated gas sensing performances of the fabricated sensors were tested on a CGS-4TPs gas sensing analysis system (Beijing Elite Tech Co., Ltd., Beijing, China), during which UV light was supplied by a UV lamp (λ = 365~385 nm, 4 W, 220 V). The vertical distance between UV lamp and sensor was 8.30 cm. In order to obtain a required concentration of target gas, a static gas distribution method was applied. The sensor response was defined as R_a_/R_g_, where R_a_ and R_g_ were the resistance of sensor in air and in target gas, respectively. The response and recover times were defined as the time required for the sensors’ resistance to 90% of the equilibrium state value after injecting and removing the target gas. During the gas sensing tests, the relative humidity (RH) in the test chamber was 20~25%.

## 3. Results and Discussion

### 3.1. Morphology and Structure Characterization of the Prepared Samples

**Figure 3:**
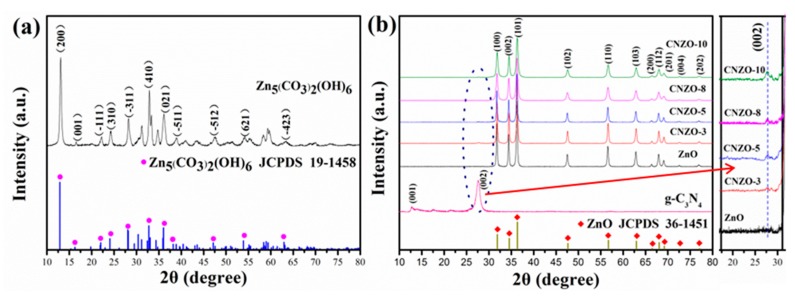
XRD patterns of the as-prepared (**a**) Zn_5_(CO_3_)_2_(OH)_6_, (**b**) g-C_3_N_4_, ZnO, CNZO-3, CNZO-5, CNZO-8, and CNZO-10.

Figure 3 shows the XRD patterns of the prepared samples. In Figure 3a, all diffraction peaks are accordance with the standard data of Zn_5_(CO_3_)_2_(OH)_6_ (JCPDS no. 19-1458), demonstrating the production of pure Zn_5_(CO_3_)_2_(OH)_6_ phase in the solvothermal step. After the annealing process, the Zn_5_(CO_3_)_2_(OH)_6_ precursor was completely transformed into hexagonal ZnO phase (JCPDS no. 36-1451), as shown in Figure 3b. The diffraction peaks at 2θ = 31.8°, 34.5°, 36.3°, 47.6°, 56.6°, 62.9°, 66.4°, 67.9°, 69.1°, 72.5° and 76.9° were indexed to (100), (002), (101), (102), (110), (103), (200), (112), (201), (400), and (202) planes of hexagonal wurtzite ZnO, respectively. In the XRD pattern of the prepared g-C_3_N_4_ sample, two distinct diffraction peaks at 13.6° and 27.9° were observed, which can be ascribed to the (100) and (002) planes of g-C_3_N_4_ [17,18], respectively. Besides of the peaks from ZnO, a small diffraction peak arising from g-C_3_N_4_ (2θ = 27.9°) was also observed in the CNZO-3, CNZO-5, CNZO-8 and CNZO-10 samples, indicating the successful preparation of the g-C_3_N_4_/ZnO composite after annealing the Zn_5_(CO_3_)_2_(OH)_6_ precursor together with g-C_3_N_4_. Moreover, the average crystallite sizes of the ZnO, CNZO-3, CNZO-5, CNZO-8 and CNZO-10 calculated by Scherrer’s formula were found to be 38.6, 33.7, 29.3, 22.8 and 21.5 nm, respectively. The decreased size of ZnO crystals with the increase of g-C_3_N_4_ contend indicates that the addition of g-C_3_N_4_ inhibited the further growth of ZnO crystals. No peaks from any other impurities were detected in all samples, showing the high purity of the obtained products. 

**Figure 4:**
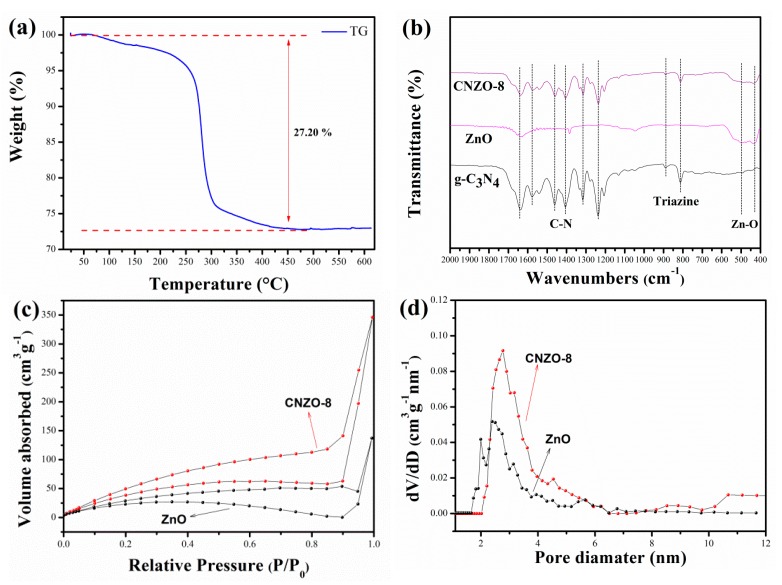
TG-DTA curves of the Zn_5(_CO_3_)_2_(OH)_6_ precursor (**a**) and FT-IR spectra (**b**) of g-C_3_N_4_, ZnO, CNZO-3, CNZO-5 and CNZO-8, Nitrogen adsorption/desorption isotherms (**c**) and pore size distribution (**d**) of ZnO and CNZO-8.

Before calcination, the thermal stability of the prepared Zn_5_(CO_3_)_2_(OH)_6_ precursor was studied. In Figure 4a, the first weight loss between 75 and 281 °C on TG curve was associated with the evaporation of adsorbed water on the sample. The second weight loss at round 281 °C corresponds a weight loss of 27.2%, which is agreement with the theoretical weight loss of Zn_5_(CO_3_)_2_(OH)_6_ decomposition to produce H_2_O, CO_2_ and ZnO. Based on above results, in our experiment, the calcination temperature was set at 400 °C to ensure the complete decomposition of the Zn_5_(CO_3_)_2_(OH)_6_ precursor. 

Figure 4b shows the FT-IR spectra of the prepared g-C_3_N_4_, ZnO and CNZO-8 samples. In the FT-IR spectrum of g-C_3_N_4_, the peaks at 813 and 890 cm^−1^ correspond to the stretching modes of triazine units [19,20], and the peaks around 1200–1650 cm^−1^ are attributed to the typical sp^2^ C=N stretching modes and the sp^3^ C-N stretching [21,22]. In the FT-IR spectrum of ZnO, the peaks at 433 and 499 cm^−1^ are ascribed to characteristic stretching of Zn–O bonds [23]. As for the composites samples of CNZO-8, besides of the characteristic peaks of Zn–O bonds, the stretching models of C-N were also detected, indicating the successful introduction of g-C_3_N_4_ in the host ZnO material.

Figure 4c,d show the N_2_ adsorption/desorption isotherms and pore size distribution of ZnO and CNZO-8, respectively. The nitrogen adsorption and desorption isotherms of both samples exhibit the type IV isotherm with H_3_ hysteresis loop. The BET surface areas of ZnO and CNZO-8 were calculated to be 95.1 and 182.1 m^2^/g, respectively. Obviously, after decoration with g-C_3_N_4_, the specific surface of ZnO hollow spheres was enlarged. Considering that the gas-sensing reaction is a surface-related process, the larger specific surface of g-C_3_N_4_/ZnO composite is helpful for achieving better gas sensing performance [24]. From the pore size distribution curves showed in Figure 4d, it can be seen that the dominant pore size in ZnO and CNZO-8 is around 2–6 nm.

**Figure 5:**
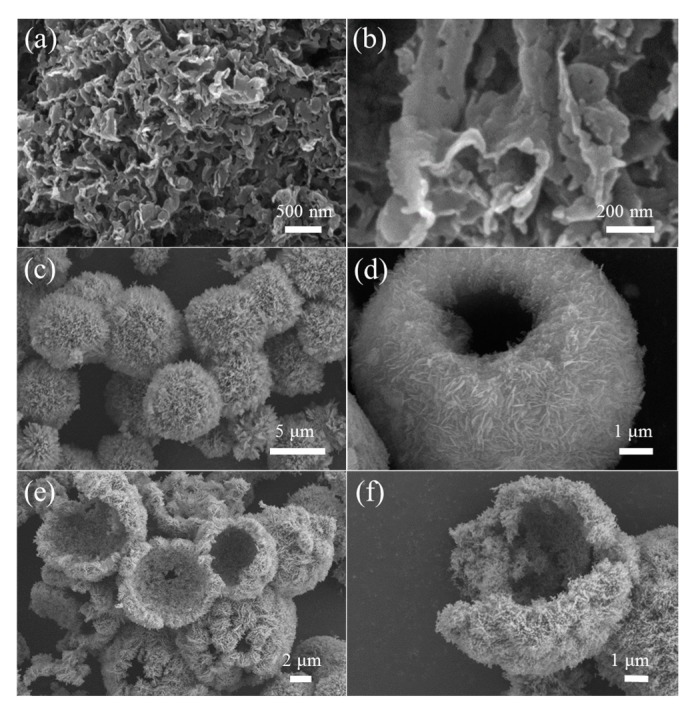
FESEM images of g-C_3_N_4_ (**a**,**b**), the precursor of CNZO-8 (**c**,**d**), and the CNZO-8 annealed at 400 °C (**e**,**f**).

**Figure 6:**
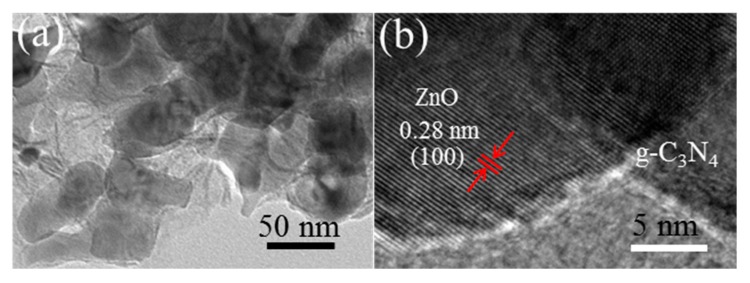
(**a**) TEM and (**b**) HRTEM images of CNZO-8.

Figure 5a,b display the typical FESEM images of the prepared g-C_3_N_4_, in which nanosheet-like g-C_3_N_4_ with the thickness about 80 nm can be clearly observed. From the low-magnification FESEM image showed in Figure 5c, one can see the prepared Zn_5_(CO_3_)_2_(OH)_6_ precursor is composed of spherical structures with the diameter about 3–5 μm. Closer observation (Figure 5d) further reveals that these microspheres are assembled from many densely stacked nanoflakes. Figure 5e,f show the FESEM images recorded from CNZO-8. It can be seen that the obtained product inherited the hollow microsphere structure from the precursor. Due to the decomposition of Zn_5_(CO_3_)_2_(OH)_6_, a loose and porous shell (about 300 nm in thickness) was formed on the ZnO hollow microspheres. In order to confirm the successful decoration of g-C_3_N_4_ nanosheets on the ZnO hollow spheres, the CNZO-8 sample was further observed by TEM. Figure 6a shows a typical TEM image recorded from the porous shell of a hollow microsphere, in which the ZnO nanoparticles (about 30–60 nm in size) covered with sheet-like g-C_3_N_4_ were clearly observed. In the HRTEM image showed in Figure 6b, clear lattice fringes were convinced. The interplanar spacing was measured to be 0.28 nm, matching well with the (100) plane of ZnO.

**Figure 7:**
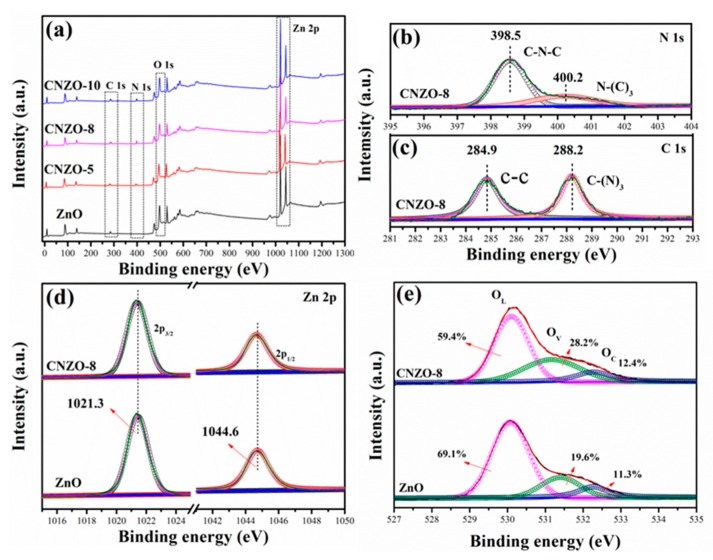
XPS survey spectra (**a**) of ZnO, CNZO-5, CNZO-8 and CNZO-10, XPS high-resolution spectrum and fitted curves of N 1s (**b**), C 1s (**c**) and Zn 2p (**d**) between ZnO and CNZO-8, O 1s (**e**) of ZnO, and CNZO-8.

The chemical composition and the chemical status of the prepared pure ZnO and g-C_3_N_4_/ZnO composites were characterized by XPS. Figure 7a shows the full-range XPS survey spectra of different samples. The sharp photoelectron peaks of Zn 2p were observed in all samples. In the composite samples, N 1s peaks with a binding energy of 398.5 and 400.2 eV were observed, but being absent in the pure ZnO sample, further demonstrating the successful introduction of g-C_3_N_4_ in ZnO. Figure 7b,c show the high-resolution N 1s and C 1s spectra of CNZO-8, respectively. In Figure 7b, the peaks at 398.5 and 400.2 eV are assigned to sp^2^-hybridized nitrogen (C–N=C) and tertiary nitrogen (N–(C)_3_), respectively [25]. In Figure 7c, the C 1s peak at 284.9 and 288.2 eV can be ascribed to graphite carbon atoms and the sp^2^-bonded carbon (N–C=N) inside the aromatic structure [26]. The Zn 2p high-resolution spectra of ZnO and CNZO-8 (Figure 7d) can be fitted into two distinct peaks, including Zn 2p_3/2_ (1021.3 eV) and 2p_2/1_ (1044.6 eV), which are in agreement with the reported value of ZnO [27]. In the high-resolution O 1s spectra (Figure 7e), the signal of O 1s can be separated into three different peaks of lattice oxygen (O_L_: 530.1 ± 0.3 eV), oxygen-deficient regions (O_V_: 531.6 ± 0.4 eV) and chemisorbed oxygen species (O_C_: 532.3 ± 0.3 eV) [28]. The relative percentages of the peaks (O_V_) in the samples of ZnO and CNZO-8 were found to be 19.6% and 28.2%, respectively. Clearly, the O_V_ component in the composite samples slightly increased with the increase of g-C_3_N_4_ content.

### 3.2. Gas Sensing Properties

**Figure 8:**
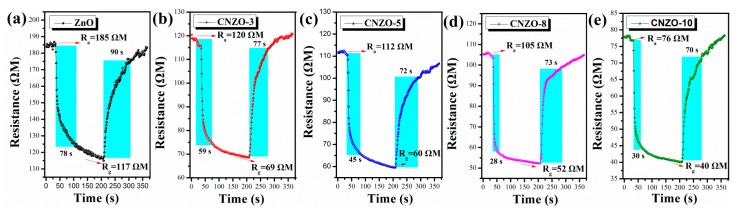
UV-light activated response resistance (**a**–**e**) curves of the sensors to 2000 ppm CH_4_ at room temperature.

The CH_4_ sensing properties of the sensors were tested under UV-light (365~385 nm) illumination at room temperature. Figure 8 shows the transient resistance change of the ZnO, CNZO-3, CNZO-5, CNZO-8 and CNZO-10 sensors as they were switched from air to CH_4_ atmosphere. It can be seen that all sensors give a decreased resistance in CH_4_, exhibiting a characteristic response of n-type MOS [29]. Importantly, as they were exposed to the same concentration of CH_4_ (2000 ppm), the five sensors showed different response-recover speeds. The measured response/recover times (Tres/Trec) were 78/90 s, 58/77 s, 45/72 s, 28/73 s and 30/70 s for ZnO, CNZO-3, CNZO-5, CNZO-8 and CNZO-10, respectively. Apparently, all the g-C_3_N_4_/ZnO sensors show faster response speed than the pure ZnO sensor. Moreover, the resistance baseline of sensors based on g-C_3_N_4_/ZnO is lower than that of the pure ZnO sensor, and decreased with increasing the amount of g-C_3_N_4_ in the composites.

**Figure 9:**
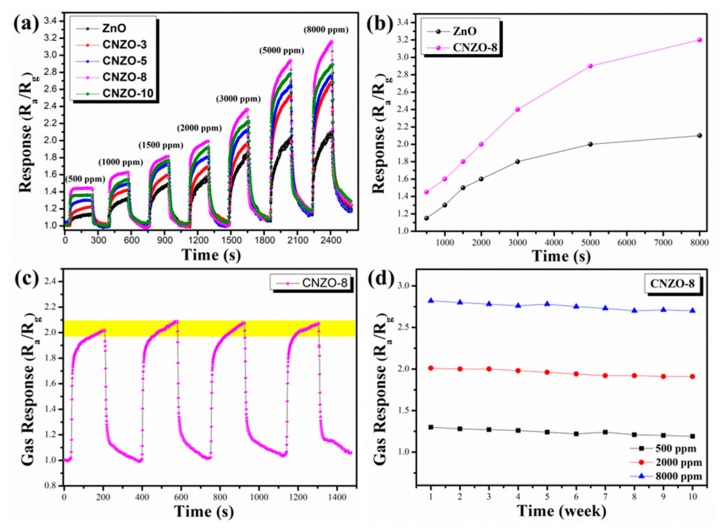
(**a**) UV light-activated dynamic response-recover curves of the g-C_3_N_4_/ZnO sensors to different concentrations of CH_4_ at room temperature, (**b**) gas responses of two sensors as a function of the ethanol concentration at room temperature, (**c**) repeatability measurements of the CNZO-8 sensor towards 2000 ppm CH_4_ at room temperature, (**d**) long-term stability test of CNZO-8 sensor to three concentrations of CH_4_ at room temperature.

The typical response curves of ZnO, CNZO-3, CNZO-5, CNZO-8 and CNZO-10 sensors at room temperature to different concentrations (500~8000 ppm) of CH_4_ with UV light activation are shown in Figure 9a. One can see that once exposed to different concentrations of CH_4_, all sensors can give a fast response before reaching their basic saturated states, and then decrease gradually as they were exposed to air again. In addition, with the increase of CH_4_ concentration, the response amplitudes of all sensors increased correspondingly, and the sensors based g-C_3_N_4_/ZnO can always give higher response amplitudes than the pure ZnO sensor, demonstrating the sensitization effect of g-C_3_N_4_ on ZnO. Among the five composite sensors, the CNZO-8 sensor shows the highest response amplitude, suggesting that the optimal content of g-C_3_N_4_ in the present composite system is 8 wt.%. Figure 9b shows the liner relationship between response and CH_4_ concentration of two different sensors. At a gas concentration of 2000 ppm, CNZO-8 sensors exhibit strong response signals (the response value is about 2 for CNZO-8 sensor). Furthermore, we can find that the sensors based on g-C_3_N_4_/ZnO have a good linear relationship in the test range (500~5000 ppm).

For practical application, good stability is an important evaluation criterion for a successful sensor. Thus, the stability of the CNZO-8 sensor was checked by testing its responses to 500, 2000, and 8000 ppm CH_4_. Figure 9c shows the repeatability test for the CNZO-8 sensor. Clearly, the CNZO-8 sensor accurately completed four consecutive gas response processes within 1500 s, and the response was basically stable at around 2. In general, sensors operating at a lower temperature (such as room temperature) can get a better stability because the higher operating temperature will affect the crystal structure of MOS sensor material. Moreover, to estimate the long-term stability of the CNZO-8 sensor, the CNZO-8 sensor exhibits a stable response to three concentrations of CH_4_ within ten weeks, in Figure 9d. It can be observed that the CNZO-8 sensor maintain its original response amplitude without significant attenuation.

### 3.3. Gas Sensing Mechanism

**Figure 10:**
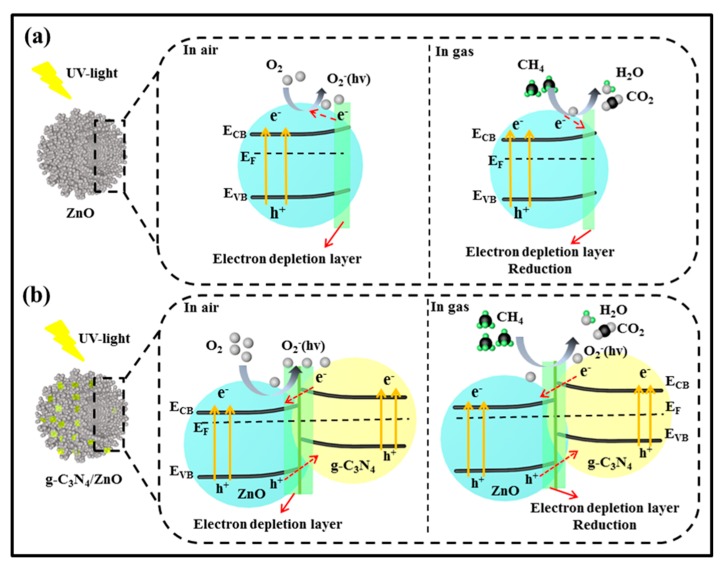
Schematic illustration for the mechanism of photoinduced charge carrier transfers in g-C3N4/ZnO composites under UV irradiation, gas sensing mechanism of (**a**) ZnO and (**b**) g-C_3_N_4_/ZnO.

The widely accepted gas sensing mechanism of MOS sensors is based on the resistance change when the sensor was switched from air to target gas, which is closely related with gas adsorption, desorption and chemical reaction on surface of the sensing materials [30]. ZnO is a typical n-type semiconductor, and the charge carriers in its conduction band (E_CB_) are dominated by electrons. As shown in Figure 10a, when the ZnO sensor is irradiated with UV light, electrons in ZnO will be excited from the valence band (E_VB_) to the E_CB_ when the energy of excitation source is higher than its band gap energy (E_g_), leaving a hole (h^+^) behind. The photo-generated electrons and holes can recombine and get trapped in metastable surface states, or react with electron acceptors and donors adsorbed on the MOS surface, respectively [31]. During the gas sensing process, when the ZnO sensors are exposed to air, O_2_ will adsorb on the surface of ZnO and capture photo-generated electron to form photoinduced oxygen ions [O_2_^−^(hv)] [32,33], resulting in a higher sensor resistance (R_a_) due to the formation of an electron depletion layer (EDL) on surface of ZnO. While, once the ZnO sensor is exposed to CH_4_, the redox reaction between the O_2_^-^(hv) and CH_4_ molecules will occur on the surface of ZnO nanoparticles, after which the trapped electrons by oxygen anions will be released back to ZnO. As a result, a lower sensor resistance (R_g_) will be obtained because of the remarkably decreased EDL thickness. Since the response of the ZnO sensor to reducing gas is generally defined as R_a_/R_g_, the different resistance values of ZnO endows its sensing ability to CH_4_.

In our experiment, it was found that after decorating with g-C_3_N_4_, the ZnO sensor showed an improved response to CH_4_ under UV-light irradiation, which may be mainly attributed to effects of g-C_3_N_4_/ZnO heterojunction. The generation of O_2_^−^ (hv) and oxygen defects will generate on the surface of heterojunction. On one hand, the formation of g-C_3_N_4_/ZnO heterojunction can promote the separation of electron-hole pair. In fact, the survival time of electron-hole pairs generated by UV-light irradiation is far shorter than the time required for migration towards the surface of the material for reacting with adsorbed oxygen ions. As a result, electron-hole recombination is a main factor that limits efficiency of the light-assisted gas sensor [34,35]. Here, the coupling effects of g-C_3_N_4_/ZnO heterostructure will take place, and some free electrons can transfer from g-C_3_N_4_ to ZnO until the two systems attain a new equilibrium Fermi energy level (E_F_), which will prevent the recombination of photo-generated electrons and holes, as illustrated in Figure 10b. Thus, during the gas sensing process, more photo-generated electrons will migrate to the surface of ZnO and react with oxygen molecules to form O_2_^−^(hv) [36]. As a result, a larger resistance change can be obtained when the sensor is switched from air to CH_4_ atmosphere, and a higher response was obtained correspondingly. Such process can be simply described as follows:

hv = h^+^ + e^−^(hv), (1)

O_2_(gas) + e^−^(hv) = O_2_^−^(ads)(hv), (2)

CH_4_ + 2O_2_^−^(ads)(hv) = CO_2_ + 2H_2_O +2e^−fig^ (3)

On the other hand, the formation of g-C_3_N_4_/ZnO heterojunction can result in the creation of more defects due to the mismatch of crystal lattice between ZnO and g-C_3_N_4_, such as oxygen vacancies O_V_. Based on the result of XPS analysis (Figure 5e), the O_V_ content in CNZO-8 (28.2%) is found to be higher than that in pure ZnO (19.6%). Xue et al. studied the double defects (Zn_i_ and O_V_) of ZnO nanodishes for ethanol sensing characteristics, which explained that the mechanism of surface defects work on gas sensing performance. The sensing performance of ZnO sensor could be promoted by its rich electron donors O_V_, resulting in that oxygen molecules are more likely to capture electrons to form O_2_^−^(hv) on ZnO [37]. Thus, on the surface of CNZO-8, more active sites can be supplied for CH_4_ adsorption and O_2_^−^(hv) creation due to its higher O_V_ content. Accordingly, when the CNZO-8 was exposed to CH_4_ atmosphere, more CH_4_ molecules will react with O_2_^−^(hv), also leading to a higher response. In addition, the higher surface area of CNZO-8 is also considered to be an important factor for its higher CH_4_ response. The result of N_2_ adsorption-desorption analysis shows that the BET surface area of CNZO-8 is 182.1 m^2^/g, much higher than that of pure ZnO (95.1 m^2^/g). Abundant previous researches have demonstrated that the higher surface area of gas sensing materials is helpful for achieving better gas sensitivity because higher surface area means that during the gas sensing process, more active sites for gas adsorption and surface reaction can be provided [38].

## 4. Conclusions

In summary, Zn_5_(CO_3_)_2_(OH)_6_ porous hollow microspheres (PHMSs) were successfully synthesized via a facile solvothermal method. By annealing the prepared Zn_5_(CO_3_)_2_(OH)_6_ PHMSs together with g-C_3_N_4_, g-C_3_N_4_ nanosheet-decorated ZnO PHMSs were successfully prepared. The results of gas sensing tests indicated that under UV-light irradiation, the sensor based on g-C_3_N_4_/ZnO PHMSs showed an improved CH_4_ sensing property than the sensor based on pure ZnO PHMSs at room temperature. The improved UV-light activated CH_4_ sensing properties of g-C_3_N_4_/ZnO PHMSs can be mainly attributed to the effects of g-C_3_N_4_-ZnO heterojunction, as well as the higher surface area of the composite materials. The formation of g-C_3_N_4_-ZnO heterojunction can not only promote the separation of electron-hole pair, but also lead to the creation of more surface oxygen vacancies, both of which can play an important role in enhancing the CH_4_ sensitivity. The present research demonstrates that decoration with g-C_3_N_4_ is a promising strategy to improve the UV-light activated CH_4_ sensing properties of ZnO.

## References

[1] Chizhov A.S., Rumyantseva M.N., Vasiliev R.B., Filatova D.G., Drozdov K.A., Krylov I.V., Abakumov A.M., Gaskov A.M. (2014). Visible light activated room temperature gas sensors based on nanocrystalline ZnO sensitized with CdSe quantum dots. Sens. Actuators B.

[2] Saboor F.H., Ueda T., Kamada K., Hyodo T., Mortazavi Y., Khodadadi A.A., Shimizu Y. (2016). Enhanced NO_2_ gas sensing performance of bare and Pd-loaded SnO_2_ thick film sensors under UV-light irradiation at room temperature. Sens. Actuators B.

[3] Lu G., Xu J., Sun J., Yu Y., Zhang Y., Liu F. (2012). UV-enhanced room temperature NO_2_ sensor using ZnO nanorods modified with SnO_2_ nanoparticles. Sens. Actuators B.

[4] Kumar A., Sanger A., Kumar A., Chandra R. (2016). Highly sensitive and selective CO gas sensor based on hydrophobic SnO_2_/CuO bilayer. RSC Adv..

[5] Hassan J.J., Mahdi M.A., Chin C.W., Abu-Hassan H., Hassan Z. (2013). A high-sensitivity room-temperature hydrogen gas sensor based on oblique and vertical ZnO nanorod arrays. Sens. Actuators B.

[6] Espid E., Taghipour F. (2017). Development of highly sensitive ZnO/In_2_O_3_ composite gas sensor activated by UV-LED. Sens. Actuators B.

[7] Gong B., Shi T., Zhu W., Liao G., Li X., Huang J., Zhou T. (2017). UV irradiation-assisted ethanol detection operated by the gas sensor based on ZnO nanowires/optical fiber hybrid structure. Sens. Actuators B.

[8] Da Silva L.F., M’Peko J.C., Catto A.C., Bernardini S., Mastelaro V.R., Aguir K., Ribeiro C., Longo E. (2016). UV-enhanced Ozone Gas Sensing Response of ZnO-SnO_2_ Heterojunctions at Room Temperature. Sens. Actuators B.

[9] Zheng Y., Liu J., Liang J., Jaroniec M., Qiao S.Z. (2012). Graphitic carbon nitride materials: Controllable synthesis and applications in fuel cells and photocatalysis. Energy Environ. Sci..

[10] Yang S.B., Gong Y.J., Zhang J.S., Zhan L., Ma L.L., Fang Z.Y., Vajtai R., Wang X.C., Ajayan P.M. (2013). Exfoliated Graphitic Carbon Nitride Nanosheets as Efficient Catalysts for Hydrogen Evolution Under Visible Light. Adv. Mater..

[11] Zhang Y., Zhang D., Guo W., Chen S. (2016). The α-Fe_2_O_3_/g-C_3_N_4_ heterostructural nanaocomposites with enhanced ethanol gas sensing performance. J. Alloys Compd..

[12] Zhai J., Wang T., Wang C., Liu D. (2018). UV-light-assisted ethanol sensing characteristics of g-C_3_N_4_/ZnO composites at room temperature. Appl. Surf. Sci..

[13] Cao S., Low J., Yu J., Jaroniec M. (2015). Polymeric Photocatalysts Based on Graphitic Carbon Nitride. Adv. Mater..

[14] Dong G., Zhang Y., Pan Q., Qiu J. (2014). A fantastic graphitic carbon nitride (g-C_3_N_4_) material: Electronic structure, photocatalytic and photoelectronic properties. J. Photochem. Photobiol. C.

[15] Geng X., Chen S., Lv X., Jiang W., Wang T. (2018). Synthesis of g-C_3_N_4_/Bi_5_O_7_I microspheres with enhanced photocatalytic activity under visible light. Appl. Surf. Sci..

[16] Cao J.L., Gong Y.X., Wang Y., Zhang B., Zhang H.L., Sun G., Hari B., Zhang Z.Y. (2017). Cocoon-like ZnO decorated graphitic carbon nitride nanocomposite: Hydrothermal synthesis and ethanol gas sensing application. Mater. Lett..

[17] Gao J., Zhou Y., Li Z., Yan S., Wang N., Zou Z. (2012). High-yield synthesis of millimetre-long, semiconducting carbon nitride nanotubes with intense photoluminescence emission and reproducible photoconductivity. Nanoscale.

[18] Thomas A., Fischer A., Goettmann F., Antonietti M., Muller J.O., Schlogl R., Carlsson J.M. (2008). Graphitic carbon nitride materials: Variation of structure and morphology and their use as metal-free catalysts. J. Mater. Chem..

[19] Zhang J., Zhang M., Zhang G., Wang X. (2012). Synthesis of Carbon Nitride Semiconductors in Sulfur Flux for Water Photoredox Catalysis. ACS Catal..

[20] Wang P., Wang Z., Jia L., Xiao Z. (2009). Origin of the catalytic activity of graphite nitride for the electrochemical reduction of oxygen: Geometric factors vs. electronic factors. Phys. Chem. Chem. Phys..

[21] Chen Y., Li J., Hong Z., Shen B., Lin B., Gao B. (2014). Origin of the enhanced visible-light photocatalytic activity of CNT modified g-C_3_N_4_ for H_2_ production. Phys. Chem. Chem. Phys..

[22] Dong F., Wu L., Sun Y., Fu M., Wu Z., Lee S.C. (2011). Efficient synthesis of polymeric g-C_3_N_4_ layered materials as novel efficient visible light driven photocatalysts. J. Mater. Chem..

[23] Raizada P., Singh P., Kumar A., Sharma G., Pare B., Jonnalagadda S.B., Thakur P. (2014). Solar photocatalytic activity of nano-ZnO supported on activated carbon or brick grain particles: Role of adsorption in dye degradation. Appl. Catal. A.

[24] Li G.J., Zhang X.H., Kawi S. (1999). Relationships between sensitivity, catalytic activity, and surface areas of SnO_2_ gas sensors. Sens. Actuators B.

[25] Fang J.W., Fan H.Q., Li M.M., Long C.B. (2015). Nitrogen self-doped graphitic carbon nitride as efficient visible light photocatalst for hydrogen evolution. J. Mater. Chem. A.

[26] Xiang Q., Yu J., Jaroniec M. (2011). Preparation and Enhanced Visible-Light Photocatalytic H_2_-Production Activity of Graphene/C_3_N_4_ Composites. J. Phys. Chem. C.

[27] Xu L., Zheng R.F., Liu S.H., Song J., Chen J.S., Dong B., Song H.W. (2012). NiO@ZnO Heterostructured Nanotubes: Coelectrospinning Fabrication, Characterization, and Highly Enhanced Gas Sensing Properties. Inorg. Chem..

[28] Alenezi M.R., Alshammari A.S., Jayawardena K.D.G.I., Beliatis M.J., Henley S.J., Silva S.R.P. (2013). Role of the Exposed Polar Facets in the Performance of Thermally and UV Activated ZnO Nanostructured Gas Sensors. J. Phys. Chem. C.

[29] Wang T., Kou X., Zhao L., Sun P., Liu C., Wang Y., Shimanoe K., Yamazoe N., Lu G. (2017). Flower-like ZnO hollow microspheres loaded with CdO nanoparticles as high performance sensing material for gas sensors. Sens. Actuators B.

[30] Park S., Sun G.J., Kheel H., Hyun S.K., Jin C., Lee C. (2016). Hydrogen gas sensing of CO_3_O_4_-Decorated WO_3_ nanowires. Met. Mater. Int..

[31] Wang J., Liu P., Fu X., Li Z., Han W., Wang X. (2008). Relationship between oxygen defects and the photocatalytic property of ZnO nanocrystals in nafion membranes. Langmuir.

[32] Soleimanpour A.M., Hou Y., Jayatissa A.H. (2011). The effect of UV irradiation on nanocrysatlline zinc oxide thin films related to gas sensing characteristics. Appl. Surf. Sci..

[33] Hyodo T., Urata K., Kamada K., Ueda T., Shimizu Y. (2017). Semiconductor-type SnO_2_-based NO_2_ sensors operated at room temperature under UV-light. Sens. Actuators, B.

[34] Tachibana Y., Vayssieres L., Durrant J.R. (2012). Artificial photosynthesis for solar water-splitting. Nat. Photonics.

[35] Hou Y., Zuo F., Dagg A.P., Liu J., Feng P. (2014). Branched WO_3_ Nanosheet Array with Layered C_3_N_4_ Heterojunctions and CoOx Nanoparticles as a Flexible Photoanode for Efficient Photoelectrochemical Water Oxidation. Adv. Mater..

[36] Fan S.W., Srivastava A.K., Dravid V.P. (2009). UV-activated room-temperature gas sensing mechanism of polycrystalline ZnO. Appl. Phys. Lett..

[37] Xue Z., Cheng Z., Xu J., Xiang Q., Wang X., Xu J. (2017). Controllable Evolution of Dual Defect Zn_i_ and V_O_ Associate-Rich ZnO Nanodishes with (0001) Exposed Facet and Its Multiple Sensitization Effect for Ethanol Detection. ACS Appl. Mater. Interfaces.

[38] Lü Y., Zhan W., He Y., Wang Y., Kong X., Kuang Q., Xie Z., Zheng L. (2014). MOF-Templated Synthesis of Porous CO_3_O_4_ Concave Nanocubes with High Specific Surface Area and Their Gas Sensing Properties. ACS Appl. Mater. Interfaces.

